# High-Performance Optically Transparent EMI Shielding Sandwich Structures Based on Irregular Aluminum Meshes: Modeling and Experiment

**DOI:** 10.3390/ma18174102

**Published:** 2025-09-01

**Authors:** Anton S. Voronin, Bogdan A. Parshin, Mstislav O. Makeev, Pavel A. Mikhalev, Yuri V. Fadeev, Fedor S. Ivanchenko, Il’ya I. Bril’, Igor A. Tambasov, Mikhail M. Simunin, Stanislav V. Khartov

**Affiliations:** 1 Regional Educational and Scientific Center “Security”, Bauman Moscow State Technical University, 105005 Moscow, Russia; parshbgal@bmstu.ru (B.A.P.); pamikhalev@bmstu.ru (P.A.M.); daf.hf@list.ru (Y.V.F.); ellaijiah@gmail.com (I.I.B.); michanel@mail.ru (M.M.S.); 2 Department of Molecular Electronics, Federal Research Center «Krasnoyarsk Science Center», Siberian Branch of the Russian Academy of Sciences (FRC KSC SB RAS), 660036 Krasnoyarsk, Russia; orion-leo@mail.ru (F.S.I.); stas_f1@list.ru (S.V.K.); 3 School of Engineering and Construction, Siberian Federal University, 660041 Krasnoyarsk, Russia; 4 Laboratory of Photonics of Molecular Systems, Kirensky Institute of Physics, Siberian Branch of the Russian Academy of Sciences, 660036 Krasnoyarsk, Russia; tambasov_igor@mail.ru; 5 LLC Research and Production Company «Spectehnauka», 660043 Krasnoyarsk, Russia

**Keywords:** cracked template, irregular aluminum mesh, electromagnetic interference shielding, sandwich structures

## Abstract

Highly efficient shielding materials, transparent in the visible and IR ranges are becoming important in practice. This stimulates the development of cheap methods for creating transparent conductors with low sheet resistance and high optical transparency. This work presents a complex approach based on preliminary modeling of the shielding characteristics of two-layer sandwich structures based on irregular aluminum mesh (IAM) formed by the cracked template method. Experimentally measured spectral dependences of the transmission coefficient of single-layer IAM are used as a reference point for modeling. According to the simulation results, two types of sandwich structures were designed using IAM, with varying filling factors and a fixed PMMA layer thickness of 4 mm. The experimentally measured shielding characteristics of the sandwich structures in the range of 0.01–7 GHz are in good agreement with the calculated data. The obtained structures demonstrate a shielding efficiency of 55.96 dB and 65.55 dB at a frequency of 3.5 GHz (the average range of 5G communications). At the same time, their optical transparency at a wavelength of 550 nm are 84.07% and 75.78%, respectively. Our sandwich structures show electromagnetic shielding performance and uniform diffraction pattern. It gives them an advantage over structures based on regular meshes. The obtained results highlight the prospect of the proposed comprehensive approach for obtaining highly efficient, low-cost optically transparent shielding structures. Such materials are needed for modern wireless communication systems and metrology applications.

## 1. Introduction

The shielding of parasitic electromagnetic fields has become increasingly important with the rapid advancement of communication technologies. The 5G communication standard is being implemented everywhere and the 6G standard is on the way. Shielding of opaque materials is a solved problem and there are a large number of technological solutions. Shielding of transparent objects is a rather complicated task and requires progress in materials and design methods of optically transparent electromagnetic screens [1]. In the case of single-layer structures, it is possible to achieve a shielding coefficient of more than 40 dB over a wide frequency range using meshes with a small cell size. They are obtained by photolithography [2,3], imprinting [4,5], and methods-based self–organization such as cracked template [6,7,8,9] and nanosphere lithography [10]. A similar result is demonstrated by metal nanofibers and nanotubes obtained by electrospinning [11,12,13]. However, in a single-layer design, a fairly significant frequency dependence is observed. Only nanomesh is capable of demonstrating a uniform broadband shielding effect, but its production is expensive and impractical for use as passive radio wave shields. Based on these considerations, it is advisable to develop cheap, reproducible methods for forming highly effective transparent coatings with a broadband effect.

To increase the shielding efficiency and achieve a broadband effect, multilayer sandwich structures in which thin films of transparent conductors alternate with dielectric spacers are usually considered. Typically, the number of layers of transparent conductors in such structures varies from two [14,15,16,17] to four [18]. In these structures, each layer makes an additive shielding contribution. The addition of dielectric spacers forms resonant cavities in which losses due to multiple reflections are observed. The main goal pursued in the process of forming shielding transparent sandwich structures is to maintain acceptable optical transmission, more than 70%, with maximum shielding efficiency; this value should be at least 50 dB and the effective range should be about 10 GHz.

Usually, the most used metals for mesh transparent conductors for shielding applications are Ag [19,20,21], Cu [22,23] and Au [24,25]. In contrast, aluminum-based transparent conductors are less used for such purposes [26], despite the fact that aluminum possesses several advantageous properties. It is the most common metal on Earth and ranks fourth in electrical conductivity, surpassed only by silver, copper, and gold. In addition, aluminum is highly resistant to atmospheric corrosion due to the formation of a thin, dense oxide film on the surface.

We have already investigated the shielding efficiency of single layer aluminum meshes of different thicknesses [26] and a sandwich structure based on silver meshes [16] in our previous works. This paper presents a comprehensive approach to the design and fabrication of highly efficient optically transparent shielding sandwich structures. The most important part of this study is the development of an analytical model that allows preliminary modeling of sandwich structures before obtaining them. This approach opens up new possibilities for targeted selection of parameters of single-layer mesh transparent conductors and effective control of their optical and shielding properties. Unlike empirical methods, this is possible without a lot of experimentation.

We use irregular aluminum mesh transparent conductors obtained by using the economical and environmentally friendly cracked template method, and manufacture high-performance shielding sandwich structures based on them with pre-predicted properties.

Thus, our integrated approach combining cost-effective and green manufacturing and advanced modeling provides a promising platform for creating high-performance optically transparent shielding sandwich structures.

## 2. Materials and Methods

### 2.1. Preparation of IAM and Sandwich Structures

**Figure 1:**
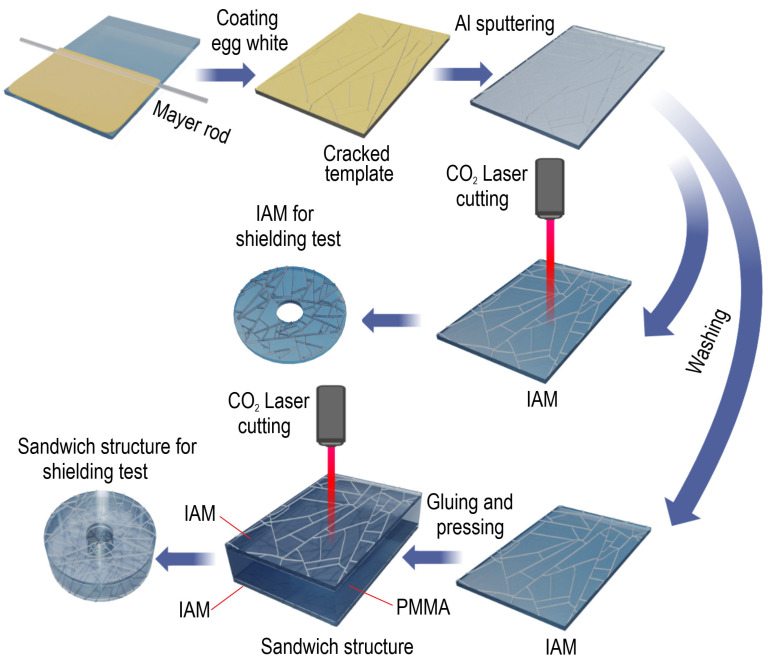
Technological scheme of IAM formation and preparation of IAM and sandwich structures based on them for measuring shielding properties.

The cracked template was formed using the liquid fraction of egg white as the base material. An additive was incorporated to induce and control the formation of surface cracks. In this work, two types of cracked template with different coefficients of surface filling with cracks were obtained. We are considering egg white solutions without additives that provoke cracking (hereinafter, the mesh coating based on this template will be called IAM №1) and with 0.01 vol.% additives that provoke cracking (the mesh coating based on this template will be called IAM №2). The complete technological process of forming IAM structures and two-layer sandwich structures with a PMMA spacer is shown in Figure 1.

Both cracked template precursors were applied by the Mayer rod method to PET substrates with a thickness of 100 µm (Hi-Fi Industrial Film Ltd., Stevenage, Hertfordshire, UK). The thickness of the precursor layer was 70.8 µm, and the application rate was 30 mm/s. The liquid film was dried at a temperature of 20 °C. During the drying of the egg white film, mechanical stresses accumulate due to shrinkage effects, which relax through cracking. This stage is the formation of the cracked template. Next, a 600 nm thick aluminum film was deposited onto the two types of cracked template by thermal sputtering. The thickness of the sputtered aluminum film was controlled using a quartz resonator. After sputtering the cracked template and excess aluminum, it was dissolved in DI water for 3 min. After washing the IAM structures, the excess DI water was blown away by an air cannon. We used square samples measuring 3 × 3 cm^2^ for the research. Further in the study, we studied both single-layer IAMs with different filling coefficients and sandwich structures based on IAMs with different filling coefficients. Using single-layer IAMs, we obtained IAM/PMMA/IAM sandwich structures as follows: 0.1 mL of photo-curing glue NOA 63 (Norland Products Inc., Jamesburg, NJ, USA) was applied to the PMMA (3 × 3 cm^2^, thickness 4 mm) spacer and evenly distributed. Then, the IAM was pressed on top of a polyethylene terephthalate substrate. A similar operation was repeated for the other face of the PMMA spacer. The resulting sandwich structure was pressed for 1 min with a force of 100 N. After pressing, without removing the pressure, they were exposed to UV radiation (20 W, λ = 365 nm) for 10 min. Then discs were cut out of the obtained sandwich structures using a CO_2_ laser (Wattsan, 1290 Duos LT, Jinan City, Shandong, China).

### 2.2. Characterizations of IAM and Sandwich Structures

The morphology of the IAM was studied using scanning electron microscopy (SEM) using Hitachi TM-4000 Plus (Hitachi, Tokyo, Japan), with the accelerating voltage at 5–20 kV. The SEM was equipped with an energy-dispersive X-ray spectrometer X-Flash 630Hc (EDX, Bruker, Billerica, MA, USA).

Optical transmittance spectra of IAM and sandwich structures were obtained in the range of 400–800 nm on a UV-3600i Plus (Shimadzu, Kyoto, Japan) spectrophotometer with an integrating sphere.

The sheet resistance of the IAM was measured by the four-probe method using a JG ST2258 four-point probe station (Suzhou Jingge Electronics Co., Suzhou, China) and a JG ST2558-F01 four-probe head (Suzhou Jingge Electronics Co., Suzhou, China).

Diffraction patterns were obtained according to the following experimental scheme: laser beam from an ATC-53–350 semiconductor laser (ATC «Semiconductor Devices», Saint-Petersburg, Russia) with a wavelength of 532 nm was incident perpendicularly on the IAM and sandwich structure, and patterns of diffracted energy distribution were obtained in a black receiving screen located at a distance of 0.5 m from the source.

The adhesive properties of the IAMs were determined using tape test method (ASTM D4541 [27]). The effect of the reusable tape test on sheet resistance of the IAMs was also studied. Scotch tape (3M, Maplewood, MN, USA) was used in all studies, and the test sample was firmly fixed on the table.

Long-term stability to external conditions was studied during a month-long exposure of IAM №1 and IAM №2 meshes at 55% humidity and room temperature, which was 21 °C. The test samples were placed in a container with the above-mentioned climatic parameters. The value of the sheet resistance of the IAMs was used as an evaluation criterion.

### 2.3. Spectroscopies of IAM and Sandwich Structure in the 0.01–7 GHz Range

In this study, transmission coefficient *S*_21_ and reflection coefficient *S*_11_ for IAM sandwich structures were measured using waveguide cells designed for different frequency ranges. Special air coaxial cell with a diameter of 16.00/6.95 mm (type II, 50 Ω). The measurements were carried out in the range of 10 MHz to 7 GHz. The measurements were carried out on a Keysight FieldFox N9916A vector network analyzer (Keysight Technologies, Santa Rosa, CA, USA). The dynamic measurement range of the *S*_21_ is 80 dB and the measurement error is not worse than  ±2 dB even on a low signal level.

## 3. Results

### 3.1. Study of Morphological and Geometrical Parameters of IAM by SEM, EDS and Statistical Analysis

**Figure 2:**
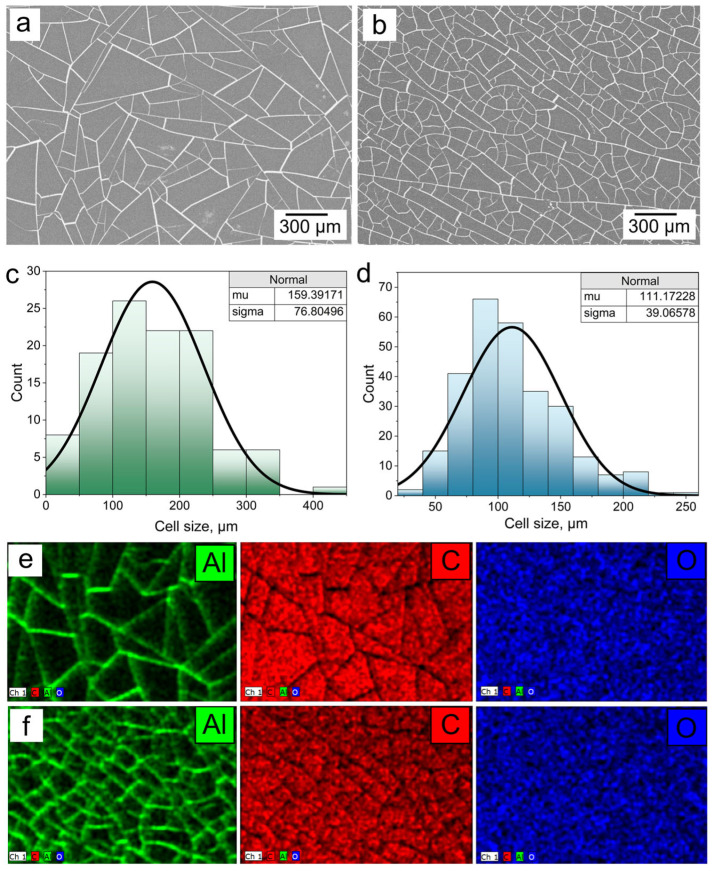
SEM images of IAM №1 (**a**) and IAM №2 (**b**); histograms of cell size distribution and their approximation by normal distribution for IAM №1 (**c**) and IAM №2 (**d**); element mapping for IAM №1 (**e**) and IAM №2 (**f**).

The geometric parameters of the IAM structures, specifically cell size, path width and Fill Factor (*FF*) are key parameters that determine optical and shielding properties. The average cell size determines the slope of the transmission spectrum in the radio frequency range [8,28], and the FF value determines the optoelectronic characteristics of IAM structures such as sheet resistance and optical transmittance. Figure 2 presents SEM images of IAM №1 (Figure 2a) and IAM №2 (Figure 2b).

Based on SEM images, the geometric characteristics of the two types of IAM structures can be estimated. By analyzing the images, it is possible to determine the pixel value of the area of each cell. Assuming a square approximation, cell size can then be calculated as $Cell\;size\sim \sqrt{S_{cell}}$, where *S_cell_* is the area of the cell. The histograms of the cell size distribution are shown in Figure 2c (IAM №1) and Figure 2d (IAM №2). Approximating the obtained histograms with a normal Poisson distribution, we obtained the following average cell sizes: 159.4 ± 76.8 µm and 6.3 ± 3.5 µm (IAM №1), and 111.2 ± 39.1 µm and 6.1 ± 2.8 µm (IAM №2). For both types of IAM, due to their stochastic structure, the standard deviation is quite significant; according to our estimates, it is 40–50% of the average cell size.

We can estimate the value of *FF* knowing the geometric parameters of the IAM. In the approximation of a mesh with square cells, the *FF* value can be calculated according to the following equation [29]:

(1) $$FF=\frac{2pw-w^{2}}{p^{2}}\;\cdot 100\%$$

where *p* is the average cell size and *w* is the average crack width. We calculated *FF* values for both types of IAM structures, which are ~7.74 ± 1.5% and ~10.67 ± 2.5%, respectively.

The inserts show the element mapping, which demonstrates that the mesh material is aluminum (Figure 2e,f). The EDX spectra for the IAM are shown in Figure S2a and S2b, respectively. Three chemical elements are present on the surface: carbon (*K_α1_* line with energy 0.277 keV) and oxygen (*K_α1_* line with energy 0.525 keV) belong to the PET substrate. Aluminum (*K_α1_* line with energy 1.486 keV) belongs to irregular mesh structures. Moreover, the intensity of the aluminum peak is quantitatively higher in IAM №2.

### 3.2. Optoelectronic Properties and Stability of IAMs

**Figure 3:**
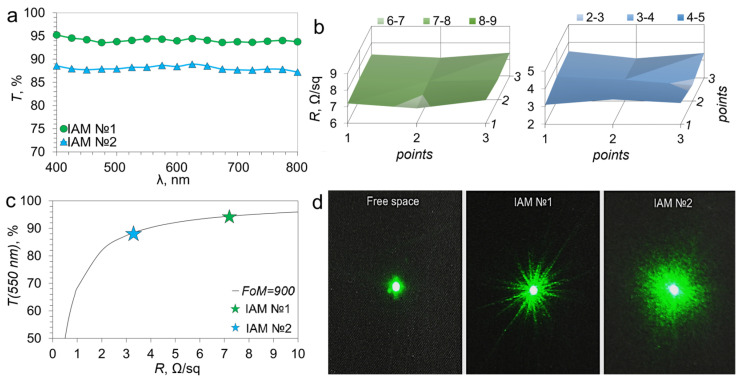
The main optoelectronic characteristics of IAM are transmission (**a**), sheet resistance (**b**), *FoM* approximation (**c**), and diffraction patterns (**d**).

The IAM transmission in the visible range is determined by the *FF* value. The transmission in the visible range can be quantified based on the equation [29]

(2) $$T=1-FF=\frac{{\left(p-w\right)}^{2}}{p^{2}}$$

where *FF* is the Fill Factor, *p* is the average period of the IAM cell, and *w* is the average width of the IAM track. According to our estimate of the fill factor above, which is 7.74 ± 1.5% (IAM №1) and 10.67 ± 2.5% (IAM №2). The estimated transmission values in the visible range are 92.26% (IAM №1) and 89.33% (IAM №2). Figure 3a shows the transmission spectra of IAM №1 and IAM №2 in the visible range. The spectra are uniform over the entire studied range. The transmission at a wavelength of 550 nm is 94.38% (IAM №1) and 88.26% (IAM №2). There is a good convergence of the estimated and experimental transmission coefficients.

It is important to understand the uniformity of the distribution of microconductors over the surface in addition to the absolute value of sheet resistance. We measured the sheet resistance of IAM structures (with an area of 9 cm^2^) at nine points. The distribution of sheet resistance for IAM №1 and IAM №2 are shown in Figure 2b. The average values of the sheet resistance are 7.2 ± 1.1 Ω/sq (IAM №1) and 3.3 ± 0.4 Ω/sq (IAM №2), which is a good result for highly transparent IAM structures.

Evaluating the relation between the values of sheet resistance and transmittance in the visible range is useful for transparent shielding materials. The relation between these two values is characterized by the figure of merit (*FoM* parameter). It can be calculated using the equation [30]

(3) $$FoM=\frac{Z_{0}}{2R(\frac{1}{\sqrt{T}}-1)}$$

where *Z*_0_ is the impedance of the free space equal to 377 Ω, *R* is the sheet resistance, and *T* is the transmission at a wavelength of 550 nm. Equation (3) approximates the experimental points for IAM №1 and IAM №2 in Figure 3c. Both types of IAM are approximated by a dependence with an *FoM* equal to 900. The obtained *FoM* value is high, surpassing Ag [31,32,33] and Cu [34] NW, as well as photolithographic [35] and imprint [36] meshes with low metallization thickness.

In addition to analyzing the transmission of visible light in the study of mesh transparent conductors, attention should be paid to the diffraction of laser radiation on the mesh structure. The features of diffraction by irregular meshes have been studied both theoretically [37] and experimentally [37,38]. Their key feature is the radially symmetric diffraction pattern resulting from the random orientation of the microconductors scattering electromagnetic waves [38]. At the same time, the diffraction of laser radiation on regular meshes, for example, square or hexagonal, has a clear connection with the symmetry of the mesh, giving figures with four- and six-ray symmetry, respectively [39]. The diffraction patterns of IAM №1 and IAM №2 are shown on Figure 3d. In the case of IAM №2, there is a clear radial blur of the laser spot, which indicates a completely random mesh geometry.

Mesh transparent conductors with geometry such as Voronoi diagrams, due to their diffraction properties, attract the attention of researchers. A number of papers have demonstrated original approaches to constructing algorithms for obtaining such meshes using photolithography [40,41] and electrohydrodynamic jet printing [42].

Adhesion strength is an important part of the characterization standards of mesh transparent conductors. We conducted two types of adhesion strength tests. First, we determined the adhesion class of each IAM according to ASTM D4541. According to the optical microscopy data (Figure S3b–e), IAM №1 and IAM №2 belong to adhesion class 5B. This means that we do not observe the delamination of the mesh elements after tearing off the adhesive tape. We can conclude that aluminum is a good choice for metallization. This is due to the fact that aluminum easily interacts with oxygen-containing functional groups that are on the surface of the PET substrate. The chemical bond provides strong adhesion of the IAM to the substrate. We also investigated the effects of 10 iterations of cyclic tape test on the sheet resistance of IAMs. Figure S3f shows the graphical dependence of the change in sheet resistance on the number of tape test cycles. A multiple tape test also does not make a visible change in the sheet resistance of IAMs.

The effect of standard conditions on the sheet resistance of IAM №1 and IAM №2 during the month is a key criterion for their practical applicability. Figure S4 shows the sheet resistance of IAM №1 and IAM №2 as a function of time. Our IAMs do not demonstrate an increase in sheet resistance under the standard conditions. We assume this is due to two factors: firstly, the formation of an oxide layer on the surface of the aluminum mesh, and secondly, the adsorption of albumin molecules (the material of the cracked template), which increase the barrier properties, under standard conditions.

### 3.3. Measurements Shielding Properties of IAMs in 0.01–7 GHz Range

The general mechanism of interaction of an electromagnetic wave propagating in a waveguide with a thin-film structure on a dielectric substrate, to which the IAMs we are studying belong, has the following form: the incident wave (*P_i_*) is decomposed into three components: transmitted (*P_t_*), reflected (*P_r_*) and absorbed (*P_a_*), from which the corresponding coefficients can be calculated, according to the equations [43]

(4) $$T=\frac{P_{t}}{P_{i}}=\left(10^{{0.1S}_{21}}\right)\cdot 100\%$$

(5) $$R=\frac{P_{r}}{P_{i}}=\left(10^{{0.1S}_{11}}\right)\cdot 100\%$$

(6) T+R+A=100%

**Figure 4:**
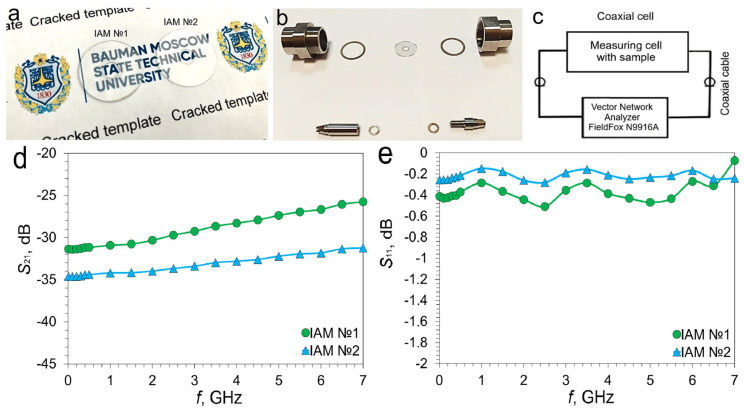
Photo IAM structures (**a**) and measuring cell (**b**); schematic representation of the measuring stand (**c**); transmission (*S*_21_) (**d**) and reflection (*S*_11_) (**e**) of IAM structures in the range 0.01–7 GHz.

Figure 4 shows a photo of IAM samples with different *FF*s (Figure 4a) and a measuring cell in a disassembled state (Figure 4b). The measurement scheme is shown in Figure 4c.

The spectral transmission for IAMs with different *FF*s in the range 0.01–7 GHz is shown in Figure 4d. The behavior of the *S*_21_ spectra for IAM №1 and IAM №2 is quite typical for metal mesh transparent conductor [8]. The frequency dependence for IAM №1 is more pronounced than for IAM №2 due to the fact that the average cell size for IAM №1 is larger. The minimum value of the *S*_21_ coefficient is observed at a frequency of 0.01 GHz and is −31.37 dB (IAM №1) and −34.62 dB (IAM №2). At 7 GHz, the bandwidth increases to −25.76 dB (IAM №1) and −31.25 dB (IAM №2), respectively. The difference of the *S*_21_ coefficients at the boundaries of the studied range is 5.61 dB and 3.37 dB, respectively.

The *S*_11_ spectra for IAM №1 and IAM №2 are shown in Figure 4e. The average reflection coefficient over the studied range is −0.37 dB (IAM №1) and −0.22 dB (IAM №2). Taking into account the recalculation according to Equation (5), in the case of IAM №1, the average reflection coefficient in the range is 91.8%; in the case of IAM №2, the reflection coefficient is 95.2%. The dominant contribution to shielding for both types of IAM is reflection. These results are typical for a mesh transparent conductor with low sheet resistance.

### 3.4. Calculation and Measurements Shielding Properties Sandwich Structures in 0.01–7 GHz Ranges

**Figure 5:**
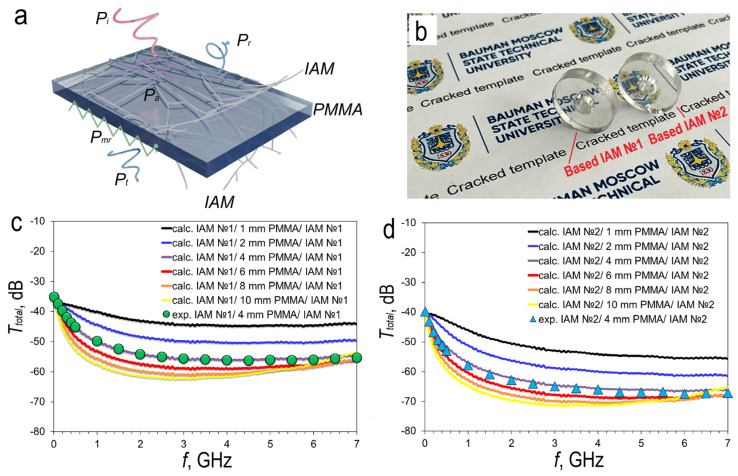
Schematic representation of the interaction of an incident electromagnetic wave with an IAM/PMMA/IAM (**a**) sandwich structure; photos of experimental sandwich structures IAM №1/4 mm PMMA/IAM №1 (left) and IAM №2/4 mm PMMA/IAM №2 (right) (**b**); calculation and experimental transmittance spectra in 0.01–7 GHz ranges for sandwich structures with different PMMA thickness: IAM №1/PMMA/IAM №1 (**c**) and IAM №2/PMMA/IAM №2 (**d**).

The mechanism of interaction of electromagnetic radiation with a sandwich structure that includes two layers of IAM and a dielectric PMMA spacer is shown in Figure 5a. In addition to the standard wave power components, which include transmitted (*P_t_*), reflected (*P_r_*), and absorbed (*P_a_*), a new component appears that is associated with the “locking” of the wave in the resonant cavity of the spacer, this component is associated with multiple reflections (*P_mr_*) [1,43].

We produced two types of sandwich structures based on two types of IAM with a PMMA spacer thickness of 4 mm to compare the results of the analytical calculation of experimental results. Photos of experimental sandwich structures of two types are shown in Figure 5b

To begin with, we will perform a model calculation, based on which we will be able to predict the spectral characteristics. Any problem of electromagnetism is based on solving the system of Maxwell’s equations [44]. The solution of Maxwell’s system of differential equations, taking into account the boundary conditions of continuity of the electric and magnetic components of the field and the material equations, is a plane wave having the following form:

(7) $$\vec{E}(z,t)={\vec{E}}_{0}e^{i(\vec{k_{z}}\cdot z-\omega t)}$$

where $\vec{k_{z}}$ is the wave vector, the projection of which onto the coordinate axis (z) is called the wavenumber and is defined as

(8) $$\vec{k_{z}}=\omega \sqrt{\varepsilon \mu }=2\pi (n+ik)/\lambda$$

where *n* is the refractive index, responsible for phase propagation, and *k* is the attenuation coefficient, responsible for exponential amplitude attenuation. This approach, based on solving the wave equation and boundary conditions on each layer, allows us to build universal algorithms for modeling multilayer structures, such as absorption dielectrics and metallic elements, taking into account both interference effects and energy loss due to absorption.

In this work, polymethylmethacrylate (PMMA) is used as a spacer, the dielectric constant dispersion of which was studied in detail in [45]. According to this study, in the range from MHz to THz, the imaginary part of the dielectric constant remains practically zero. This is due to the fact that the polarization of the spatial charge does not have time to follow the changing field. Thus, the real part of the dielectric constant is stabilized at a level $\varepsilon^{\prime }\approx 2.5$ that corresponds to the refractive index $n=\sqrt{\varepsilon^{\prime }}=\sqrt{2.5}\approx 1.6$.

In the range of 0.01–7 GHz, the thickness of the skin layer for aluminum decreases from ~26 µm to ~0.98 µm [46]. Considering that the thickness of the IAM is 600 nm, and taking into account the coefficient of transfer to the bottom of the crack [16], this value is ~400 nm. IAM structures can be considered as thin elements without thickness, with specified amplitude coefficients of reflection *S*_11_ (*f*) and transmission *S*_21_ (*f*) measured experimentally in dB. For calculations, these parameters are converted to amplitude coefficients of reflection $r=10^{S_{11}/20}$ and transmission $t=10^{S_{21}/20}$ (in absolute values), ignoring the attenuation inside the mesh and assuming that the electromagnetic wave is either reflected or transmitted. After passing through the first IAM, the wave enters a dielectric layer with a thickness of *d*, in which it propagates according to the wave propagation law (7), with a phase multiplier $e^{-i\frac{2\pi n}{\lambda }d}$ or $e^{-i\frac{2\pi n}{c}df}$. Taking into account the multiple reflections between the first and second IAM, the total amplitude transmission coefficient can be described by the following equation:

(9) $$T_{total}=\left|\frac{t^{2}e^{-i\frac{2\pi n}{c}df}}{1-r^{2}e^{-2i\frac{2\pi n}{c}df}}\right|$$

where *t* and *r* are the transmission and reflection coefficients of a single-layer IAM, *n*, *d* are the refractive index and thickness of PMMA spacer, and *c* is the speed of light in a vacuum. The transmission coefficient is a complex quantity that simultaneously contains information about both the amplitude change and the phase shift of the wave. For the analysis of the wave intensity variation during propagation through the multilayer structure, the modulus of this coefficient (*T_total_*) is considered, which corresponds to the transmitted amplitude.

We used this model to calculate the spectral dependences of the transmission coefficient. Calculations were performed for two-layer sandwich structures with different PMMA spacer thicknesses. The thickness of the spacer was 1, 2, 4, 6, 8 and 10 mm. Two configurations of sandwich structures were studied: IAM №1/PMMA/IAM №1 (Figure 5c) and IAM №2/PMMA/IAM №2 (Figure 5d).

The spectral dependences of the transmission coefficient show similar trends for the six thicknesses of the PMMA spacer in both types of sandwich structures. However, there is a difference: the transmission of sandwich structures based on IAM №2 is lower. This is due to the lower sheet resistance of each of the two layers. This fact indicates a higher shielding efficiency of such structures. The shape of the spectra is similar for both types of sandwich structures and demonstrates the same dependence on the thickness of the PMMA spacer. Therefore, the description of the behavior of transmission spectra can be generalized. In the range of 0.01–1 GHz, there is a decrease in the transmission coefficient for both types of structures. The rate of transmission reduction significantly depends on the thickness of the PMMA spacer. For the IAM №1/1 mm PMMA/IAM №1 sandwich structure, the transmission coefficient decreases from −37.23 dB (at 0.01 GHz) to −39.98 dB (at 1 GHz) (Figure 5c). For the IAM №1/10 mm PMMA/IAM №1 sandwich structure, the transmission coefficient decreases from −37.29 dB (at 0.01 GHz) to −57.31 dB (at 1 GHz). These examples demonstrate the extreme cases of the of the spectra trends, their minimum and maximum change with increasing frequency. At frequencies above 1 GHz, the reduction transmission rate is significantly reduced. This trend is observed for structures with a PMMA spacer thickness of 1, 2, and 4 mm. For structures with a PMMA thickness of 8 and 10 mm, a local minimum of transmission is observed at a frequency range of 2–3 GHz, followed by a gradual increase in the transmission coefficient. The observed decrease in the transmission coefficient in the 1–7 GHz range in sandwich structures formed by IAMs with increasing PMMA spacer thickness is caused not by monotonic field attenuation, but by interference effects in a Fabry–Perot resonator formed by the metallized surfaces. As shown in Equation (9), the phase accumulated in the PMMA spacer is directly proportional to the optical path length, $\varphi =\frac{2\pi f}{c}nd$. Therefore, increasing the spacer thickness increases the phase difference between the direct wave and the waves undergoing multiple reflections between the IAMs. This increased phase shift can cause partial destructive interference: the re-reflected waves arrive out of phase with the direct wave, reducing the overall amplitude of the transmitted wave. Figure S5 shows the dependence of the positions of the interference maximum (Figure S5a) and minimum (Figure S5b) on the PMMA spacer thickness. For most of the considered thicknesses, the first interference minimum falls within the studied frequency range (Figure S5b).

In the case of the IAM №2/1 mm PMMA/IAM №2 sandwich structure, the transmission coefficient decreases from −40.56 dB (at 0.01 GHz) to −46.05 dB (at 1 GHz) (Figure 5d). For the IAM №2/10 mm PMMA/IAM №2 sandwich structure, the transmission coefficient is reduced from −40.69 dB (at 0.01 GHz) to −64.62 dB (at 1 GHz) (Figure 5d). As in the case of the IAM №1/PMMA/IAM №1 sandwich structures, the rate of transmission reduction is significantly reduced at frequencies above 1 GHz. This pattern is typical for sandwich structures with a PMMA spacer thickness of 1, 2, 4, and 6 mm. Sandwich structures with a PMMA thickness of 8 and 10 mm have a local minimum transmission at a frequency of 3.15 GHz, followed by a gradual increase in the transmission coefficient. The minimum transmission value for structure IAM №2/10 mm PMMA/IAM №2 reaches −70.56 dB at a frequency of 3.15 GHz.

The analysis of the calculated transmission coefficient spectra prompted us to produce two types of sandwich structure with a PMMA spacer thickness of 4 mm. The calculated spectra for both types of sandwich structures with a 4 mm spacer show good agreement with experimental data, which confirms the correctness of the assumptions and simplifications adopted when constructing the model for the structures under study. At a frequency of 3.5 GHz, which belongs to the average 5G range, the transmission coefficient values of −55.96 dB were recorded for the sandwich structure IAM №1/4 mm PMMA/IAM №1 (Figure 5c, circles line) and −65.55 dB for the sandwich structure IAM №2/4 mm PMMA/IAM №2 (Figure 5d, circles line). The experimental spectra show a uniform trend in the range of 2–7 GHz, which makes it possible to characterize the obtained sandwich structures with averaged values of the transmission coefficient: −55.86 dB for the sandwich structure IAM №1/4 mm PMMA/IAM №1 and −66.04 dB for sandwich structure IAM №2/4 mm PMMA/IAM №2.

### 3.5. Optical Properties Sandwich Structures

**Figure 6:**
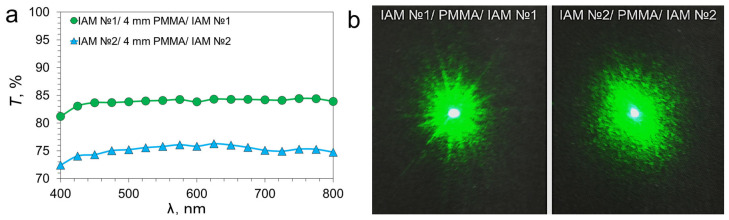
Transmission in the visible range (**a**) and diffraction patterns (**b**) of two-layer sandwich structures IAM №1/4 mm PMMA/IAM №1 and IAM №2/4 mm PMMA/IAM №2.

The optical properties of the IAM/PMMA/IAM sandwich structures differ from single IAMs in that the incident light interacts sequentially with two IAMs. Figure 6a shows the transmission spectra of two types of sandwich structures with a PMMA spacer thickness of 4 mm.

The transmission spectra of two-layer sandwich structures are a consequence of the addition of losses from two separate IAMs. At a wavelength of 550 nm, the transmission coefficient for the IAM №1/4 mm PMMA/IAM №1 sandwich structure is 84.07%, and for the IAM №2/4 mm PMMA/IAM №2 sandwich structure it is 75.78%.

The diffraction patterns for sandwich structures IAM №1/4 mm PMMA/IAM №1 and IAM №2/4 mm PMMA/IAM №2 also have radial symmetry, as in the case of single-layer IAM. Diffraction patterns show an increase in the scattering spot radius relative to single IAMs, which indicates that two consecutive IAM structures additively scatter incident laser radiation.

### 3.6. Comparison of Optical and Shielding Properties of Sandwich Structures with Literature

Comparative analysis is a convenient method for comparing different types of materials according to their basic functional properties. The shielding effectiveness (SE) of the structure can be evaluated based on the amplitude transmission coefficient *S*_21_. Since *S*_21_ is measured in decibels (dB), the shielding effectiveness is calculated as [43]

(10) $$SE=-S_{21}$$

**Figure 7:**
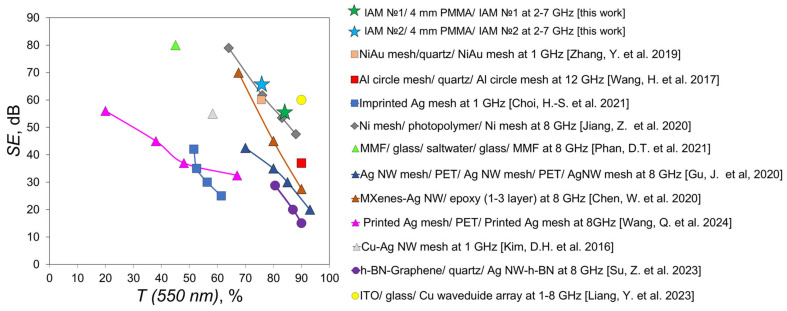
Comparison of the dependence of *SE* (*T* (*550* nm)) for our sandwich structures and the most promising literary results [18,47,48,49,50,51,52,53,54,55,56].

Using the data obtained in the experiment, we calculated the averaged SE in the range of 2–7 GHz, since the spectra in this range are fairly uniform. For the sandwich structure of IAM №1/4 mm PMMA/IAM №1, the SE value is 55.86 dB, and for the sandwich structure of IAM №2/4 mm PMMA/IAM №2 is 66.04 dB. Figure 7 shows a comparative graph in coordinates *SE* (*T* (*550* nm)).

We compared two-layer sandwich structures obtained by laser ablation [47], photolithography [48,50], and hybrid multilayer sandwich structures such as MMF/glass/saltwater/glass/MMF [51], h-BN-Graphene/quartz/Ag NW-h-BN [55] and ITO/glass/Cu waveguide array [56]. We can conclude that the sandwich structures we have obtained demonstrate comparable operational parameters with materials of a similar type obtained by more expensive methods. While Ag NW based hybrids, such as multi-layer structure MXenes-Ag NW with an epoxy resin spacer [52], have lower parameters in the two-layer design, an increase in layers leads to significantly lower transmission in the visible range.

## 4. Conclusions

We demonstrated a simple and reproducible approach for obtaining and predicting highly efficient shielding sandwich structures based on irregular aluminum meshes produced using cracked template. The model we developed demonstrates good agreement with experimental results, and can be used to predict the shielding effectiveness of multilayer shielding structures. Consequently, the proposed model provides an efficient tool for analyzing the electrodynamic behavior of multilayer systems containing metal meshes separated by a PMMA spacer. Due to its physical validity and compactness, the model demonstrates good agreement with experimental data, which makes it useful both at the design stage and for analyzing already implemented systems.

## Data Availability

The original contributions presented in this study are included in the article. Further inquiries can be directed to the corresponding author.

## Supplementary Materials

The following supporting information can be downloaded at https://www.mdpi.com/article/10.3390/ma18174102/s1, Figure S1: Examples of processed SEM images for IAM №1 and IAM №2; Figure S2: EDX spectra of IAM №1 (a) and IAM №2 (b); Figure S3: Photo IAM №1 with notch lattice according to ASTM D4541 (a); Optical microscope IAM №1 before (b) and after (c) tape test; Optical microscope IAM №2 before (d) and after (e) tape test; Influence of tape test cycles on sheet resistance of IAM №1 and IAM №2 (f); Figure S4: IAM №1 and IAM №2 stability at standard condition; Figure S5: Dependence of the interference maximum (a) and minimum (b) frequency of the studied sandwich structures on the thickness of the PMMA spacer.

## Abbreviations

The following abbreviations are used in this manuscript:

IAM	Irregular aluminum mesh
SEM	Scanning electron microscopy
*FF*	Fill factor
EMI	Electromagnetic interference
PMMA	Polymethylmetacryalte
PET	Polyetheleneterephtalate

## References

[1] Tan D., Jiang C., Li Q., Bi S., Wang X., Song J. (2021). Development and current situation of flexible and transparent EM shielding materials. J. Mater. Sci. Mater. Electron..

[2] Liang Z., Zhao Z., Pu M., Luo J., Xie X., Wang Y., Guo Y., Ma X., Luo X. (2020). Metallic nanomesh for high-performance transparent electromagnetic shielding. Opt. Mater. Express.

[3] Osipkov A., Makeev M., Konopleva E., Kudrina N., Gorobinskiy L., Mikhalev P., Ryzhenko D., Yurkov G. (2021). Optically transparent and highly conductive electrodes for acousto-optical devices. Materials.

[4] Chung S.-I., Kim P.K., Ha T.-G., Han J.T. (2019). High-performance flexible transparent nanomesh electrodes. Nanotechnology.

[5] Chung S.-I., Kim P.K., Ha T.-G. (2023). High-performance transparent electromagnetic interference shielding film based on metal meshes. J. Micromech. Microeng..

[6] Han Y., Liu Y.X., Han L., Lin J., Jin P. (2017). High-performance hierarchical graphene/metal-mesh film for optically transparent electromagnetic interference shielding. Carbon.

[7] Voronin A.S., Fadeev Y.V., Govorun I.V., Podshivalov I.V., Simunin M.M., Tambasov I.A., Karpova D.V., Smolyarova T.E., Lukyanenko A.V., Karacharov A.A. (2021). Cu–Ag and Ni–Ag meshes based on cracked template as efficient transparent electromagnetic shielding coating with excellent mechanical performance. J. Mater. Sci..

[8] Voronin A.S., Fadeev Y.V., Makeev M.O., Mikhalev P.A., Osipkov A.S., Provatorov A.S., Ryzhenko D.S., Yurkov G.Y., Simunin M.M., Karpova D.V. (2022). Low Cost Embedded Copper Mesh Based on Cracked Template for Highly Durability Transparent EMI Shielding Films. Materials.

[9] Guan Y., Yang L., Chen C., Wan R., Guo C., Wang P. (2025). Regulable crack patterns for the fabrication of high-performance transparent EMI shielding windows. iScience.

[10] Zarei M., Mohammadi K., Mahmood A.A., Li M., Leu P.W. (2025). Flexible embedded metal meshes by nanosphere lithography for very low sheet resistance transparent electrodes, Joule heating, and electromagnetic interference shielding. ACS Appl. Electron. Mater..

[11] Lin S., Wang H., Wu F., Wang Q., Bai X., Zu D., Song J., Wang D., Liu Z., Li Z. (2019). Room-temperature production of silver-nanofiber film for large-area, transparent and flexible surface electromagnetic interference shielding. NPJ Flex. Electron..

[12] Yang Y., Chen S., Li W., Li P., Ma J., Li B., Zhao X., Ju Z., Chang H., Xiao L. (2020). Reduced graphene oxide conformally wrapped silver nanowire networks for flexible transparent heating and electromagnetic interference shielding. ACS Nano.

[13] Jiang C., Tan D., Li Q., Huang J., Bu J., Zang L., Ji R., Bi S., Guo Q. (2021). High-performance and reliable silver nanotube networks for efficient and large-scale transparent electromagnetic interference shielding. ACS Appl. Mater. Interfaces.

[14] Liao D., Zhou J., Zheng Y. (2025). Preparation and performance of double-layer metal mesh transparent conductive films based on crack template method. Acta Phys. Sin..

[15] Zarei M., Li M., Papazekos E., Su Y.-D., Sinha S., Walker S.B., LeMieux M., Ohodnicki P.R., Leu P.W. (2024). Single- and double-layer embedded metal meshes for flexible, highly transparent electromagnetic interference shielding. Adv. Mater. Technol..

[16] Voronin A.S., Fadeev Y.V., Ivanchenko F.S., Dobrosmyslov S.S., Makeev M.O., Mikhalev P.A., Osipkov A.S., Damaratsky I.A., Ryzhenko D.S., Yurkov G.Y. (2023). Original concept of cracked template with controlled peeling of the cells perimeter for high performance transparent EMI shielding films. Surf. Interfaces.

[17] Chen Q., Huang L., Wang X., Yuan Y. (2023). Transparent and flexible composite films with excellent electromagnetic interference shielding and thermal insulating performance. ACS Appl. Mater. Interfaces.

[18] Gu J., Hu S., Ji H., Feng H., Zhao W., Wei J., Li M. (2020). Multi-layer silver nanowire/polyethylene terephthalate mesh structure for highly efficient transparent electromagnetic interference shielding. Nanotechnology.

[19] Lei Q., Luo Z., Zheng X., Lu N., Zhang Y., Huang J., Yang L., Gao S., Liang Y., He S. (2023). Broadband transparent and flexible silver mesh for efficient electromagnetic interference shielding and high-quality free-space optical communication. Opt. Mater. Express.

[20] Kim M.-H., Joh H., Hong S.-H., Oh S.J. (2019). Coupled Ag nanocrystal-based transparent mesh electrodes for transparent and flexible electro-magnetic interference shielding films. Curr. Appl. Phys..

[21] Voronin A.S., Fadeev Y.V., Govorun I.V., Voloshin A.S., Tambasov I.A., Simunin M.M., Khartov S.V. (2021). A transparent radio frequency shielding coating obtained using a self-organized template. Tech. Phys. Lett..

[22] Tai Y., Zhou J., Zhu X., Zhang H., Li H., Li Z., Wang R., Zhang F., Zhang G., Liu C. (2023). Additive manufacturing of large-scale metal mesh with core-shell composite structure for transparent electromagnetic shielding/glass heater. Chin. J. Mech. Eng. Addit. Manuf. Front..

[23] Li T., Chen X., Xu Z., Nie S., Xu W., Yuan W., Xu S., Zhang S., Pei F., Su W. (2025). High-performance visible-infrared broadband transparent copper mesh conductor and applications for electromagnetic shielding and heating. Sci. China Mater..

[24] Yang L., Guo R., Gao F., Guan Y., Zhang M., Wang P. (2025). Electromagnetic interference (EMI) shielding performance and photoelectric characteristics of ZnS Infrared Window. Materials.

[25] Liang Y., Huang X., Wen K., Wu Z., Yao L., Pan J., Liu W., Liu P. (2023). Metal mesh-based infrared transparent EMI shielding window with balanced shielding properties over a wide frequency spectrum. Appl. Sci..

[26] Voronin A.S., Makeev M.O., Damaratsky I.A. Aluminium mesh transparent conductor with irregular structure as effective EMI shielding material // IEEEXplore. Proceedings of the 2024 International Ural Conference on Electrical Power Engineering (UralCon).

[27] (2022). Standard Test Method for Pull-Off Strength of Coatings Using Portable Adhesion Testers.

[28] Khodzitsky M.K., Bassarab V.V., Shakhmin A.A., Sokolov V.S., Kropotov G.I. (2021). The electromagnetic shielding of optoelectronic devices by mesh structures. Appl. Sci..

[29] Lee H.B., Jin W.-Y., Ovhal M.M., Kumar N., Kang J.-W. (2019). Flexible transparent conducting electrodes based on metal meshes for organic optoelectronic device applications: A review. J. Mater. Chem. C.

[30] Ellmer K. (2012). Past achievements and future challenges in the development of optically transparent electrodes. Nat. Photon..

[31] Qin F., Yan Z., Fan J., Cai J., Zhu X., Zhang X. (2021). Highly uniform and stable transparent electromagnetic interference shielding film based on silver nanowire–PEDOT:PSS composite for high power microwave shielding. Macromol. Mater. Eng..

[32] Zhu X., Xu J., Qin F., Yan Z., Guo A., Kan C. (2020). Highly efficient and stable transparent electromagnetic interference shielding films based on silver nanowires. Nanoscale.

[33] Voronin A., Bril’ I., Pavlikov A., Makeev M., Mikhalev P., Parshin B., Fadeev Y., Khodzitsky M., Simunin M., Khartov S. (2025). THz shielding properties of optically transparent PEDOT:PSS/AgNW composite films and their sandwich structures. Polymers.

[34] Guo H., Lin N., Chen Y., Wang Z., Xie Q., Zheng T., Gao N., Li S., Kang J., Cai D. (2013). Copper nanowires as fully transparent conductive electrodes. Sci. Rep..

[35] Kim W.-K., Lee S., Lee D.H., Park I.H., Bae J.S., Lee T.W., Kim J.-Y., Park J.H., Cho Y.C., Cho C.R. (2015). Cu mesh for flexible transparent conductive electrode. Sci. Rep..

[36] Kim H.-J., Kim Y., Jeong J.-H., Choi J.-H., Lee J., Choi D.-G. (2015). Cupronickel-based micromesh film for use as a high-performance and low-voltage transparent heater. J. Mater. Chem. A.

[37] Liao D., Kang L., Zhou J. (2025). Calculation and verification of optical diffraction performance of random metallic meshes. J. Appl. Phys..

[38] Han Y., Lin J., Liu Y., Fu H., Ma Y., Jin P., Tan J. (2016). Crackle template based metallic mesh with highly homogeneous light transmission for high-performance transparent EMI shielding. Sci. Rep..

[39] Halman J.I., Ramsey K.A., Thomas M., Griffin A. Predicted and measured transmission and diffraction by a metallic mesh coating. Proceedings of the Window and Dome Technologies and Materials XI.

[40] Zhong H., Han Y., Lin J., Jin P. (2020). Pattern randomization: An efficient way to design high-performance metallic meshes with uniform stray light for EMI shielding. Opt. Express.

[41] Liao D., Zheng Y., Ma X., Fu Y. (2023). Honeycomb-ring hybrid random mesh design with electromagnetic interference (EMI) shielding for low stray light. Opt. Express.

[42] Cao D., Ma J., Li C., Guan Y., Hu J., Feng J., Wang L., Wang Y., Lin J., Jin P. (2025). Voronoi diagrams metallic mesh for transparent EMI shielding. Appl. Phys. Lett..

[43] Voronin A.S., Fadeev Y.V., Ivanchenko F.S., Dobrosmyslov S.S., Simunin M.M., Govorun I.V., Podshivalov I.V., Mikhalev P.A., Makeev M.O., Damaratskiy I.A. (2025). Waste-free self-organized process manufacturing transparent conductive mesh and micro flakes in closed cycle for broadband electromagnetic shielding and heater application. J. Mater. Sci. Mater. Electron..

[44] Landau L.D., Pitaevskii L.P., Lifshitz E.M. (1984). Electrodynamics of continuous media. Course of Theoretical Physics.

[45] Shi Z., Song L., Zhang T. (2019). Optical and electrical characterization of pure PMMA for terahertz wide-band metamaterial absorbers. J. Infrared Millim. Terahertz Waves.

[46] Hassan S.G., Mohammad E.J. (2021). Study of the skin depth in different metallic atoms. Mater. Today Proceed..

[47] Zhang Y., Dong H., Li Q., Mou N., Chen L., Zhang L. (2019). Double-layer metal mesh etched by femtosecond laser for high-performance electromagnetic interference shielding window. RSC Adv..

[48] Wang H., Lu Z., Liu Y., Tan J., Ma L., Lin S. (2017). Double-layer interlaced nested multi-ring array metallic mesh for high-performance transparent electromagnetic interference shielding. Opt. Lett..

[49] Choi H.-S., Suh S.-J., Kim S.-W., Kim H.-J., Park J.-W. (2021). Transparent electromagnetic shielding film utilizing imprinting-based micro patterning technology. Polymers.

[50] Jiang Z., Zhao S., Huang W., Chen L., Liu Y.-H. (2020). Embedded flexible and transparent double-layer nickel-mesh for high shielding efficiency. Opt. Express.

[51] Phan D.T., Jung C.W. (2021). Optically transparent and very thin structure against electromagnetic pulse (EMP) using metal mesh and saltwater for shielding windows. Sci. Rep..

[52] Chen W., Liu L.-X., Zhang H.-B., Yu Z.-Z. (2020). Flexible, transparent, and conductive Ti_3_C_2_T_x_ MXene−silver nanowire films with smart acoustic sensitivity for high-performance electromagnetic interference shielding. ACS Nano.

[53] Wang Q., Feng Y., Lin F., Chen Y., Ding N., Zhang Y., Liu S., Zhao W., Zhao Q. (2024). High-precision printing sandwich flexible transparent silver mesh for tunable electromagnetic interference shielding visualization windows. ACS Appl. Mater. Interfaces.

[54] Kim D.-H., Kim Y., Kim J.-W. (2016). Transparent and flexible film for shielding electromagnetic interference. Mater. Design.

[55] Su Z., Yang H., Wang G., Zhang Y., Zhang J., Lin J., Jia D., Wang H., Lu Z., Hu P.A. (2023). Transparent and high-performance electromagnetic interference shielding composite film based on single-crystal graphene/hexagonal boron nitride heterostructure. J. Colloid Interface Sci..

[56] Liang Y., Huang X., Pan J., Liu W., Wen K., Zhai D., Shang P., Liu P. (2023). Shorted micro-waveguide array for high optical transparency and superior electromagnetic shielding in ultra-wideband frequency spectrum. Adv. Mater. Technol..

