# Supplementary material for: High-Performance Optically Transparent EMI Shielding Sandwich Structures Based on Irregular Aluminum Meshes: Modeling and Experiment
Source: Materials (Basel). 2025 Sep 1;18(17):4102. doi: 10.3390/ma18174102 (PMC12430446; doi:10.3390/ma18174102)
Supplement: Supplementary file 1 [file materials-18-04102-s001.zip › materials-3824106-supplementary.pdf]

materials

MDPI

High-Performance Optically Transparent EMI Shielding Sandwich Structures Based on Irregular Aluminum Meshes: Modeling and Experiment

Anton S. Voronin [1,2,3,*], Bogdan A. Parshin [1], Mstislav O. Makeev [1,*], Pavel A. Mikhalev [1], Yuri V. Fadeev [1,2], Fedor S. Ivanchenko [2], Il'ya I. Bril' [1,2], Igor A. Tambasov [4,5], Mikhail M. Simunin [1,2,3] and Stanislav V. Khartov [2]

1 Regional Educational and Scientific Center "Security", Bauman Moscow State Technical University, 105005 Moscow, Russia; parshbgal@bmstu.ru (B.A.P.); pamikhalev@bmstu.ru (P.A.M.); daf.hf@list.ru (Y.V.F.); ellaijiah@gmail.com (I.I.B.); michanel@mail.ru (M.M.S.)
2 Department of Molecular Electronics, Federal Research Center «Krasnoyarsk Science Center», Siberian Branch of the Russian Academy of Sciences (FRC KSC SB RAS), 660036 Krasnoyarsk, Russia; orion-leo@mail.ru (F.S.I.); stas_f1@list.ru (S.V.K.)
3 School of Engineering and Construction, Siberian Federal University, 660041 Krasnoyarsk, Russia
4 Laboratory of Photonics of Molecular Systems, Kirensky Institute of Physics, Siberian Branch of the Russian Academy of Sciences, 660036 Krasnoyarsk, Russia; tambasov_igor@mail.ru
5 LLC Research and Production Company «Spectehnauka», 660043 Krasnoyarsk, Russia
* Correspondence: a.voronin1988@mail.ru (A.S.V.); m.makeev@bmstu.ru (M.O.M.)

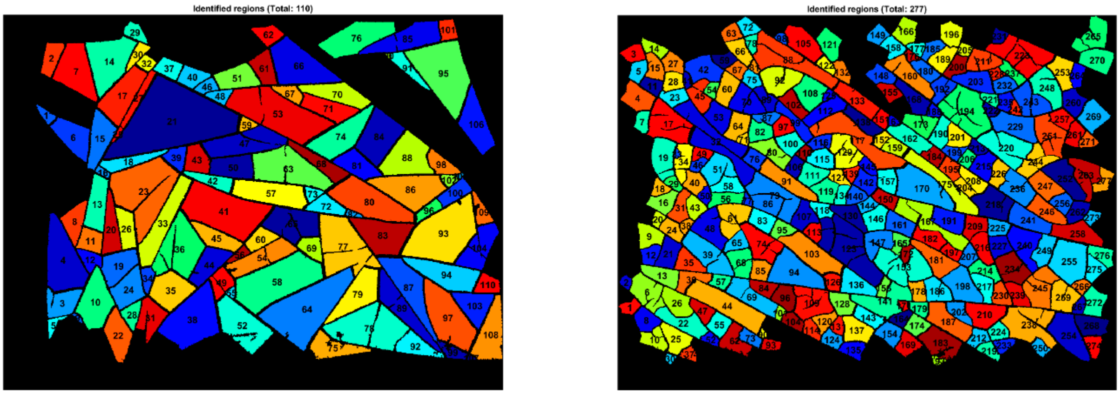

Figure S1 Examples of processed SEM images for IAM №1 and IAM №2

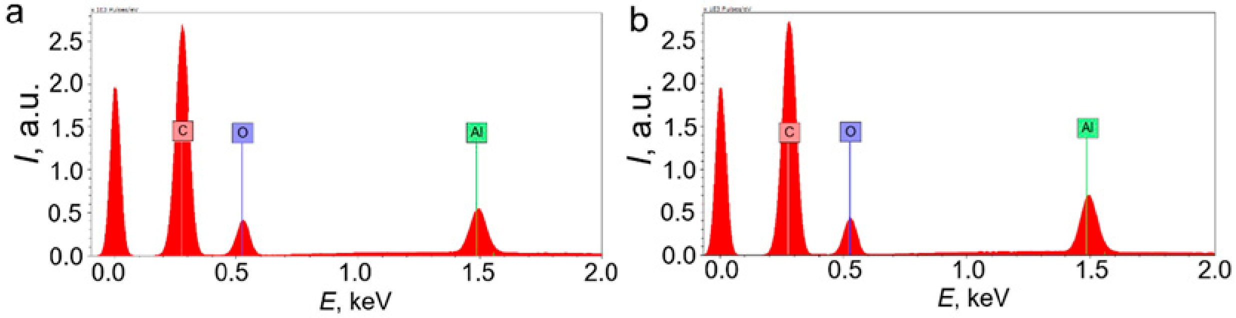

Figure S2 EDX spectra of IAM №1 (a) and IAM №2 (b)

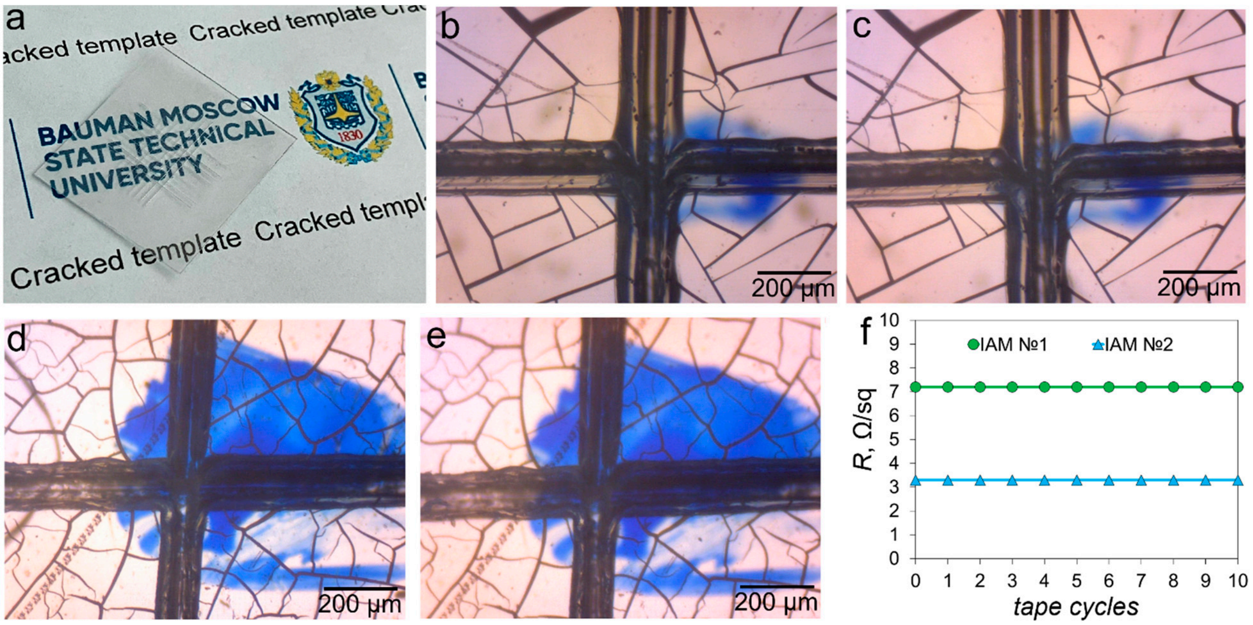

Figure S3 Photo IAM №1 with notch lattice according to ASTM D4541 (a); Optical microscope IAM №1 before (b) and after (c) tape test; Optical microscope IAM №2 before (d) and after (e) tape test; Influence of tape test cycles on sheet resistance of IAM №1 and IAM №2 (f)

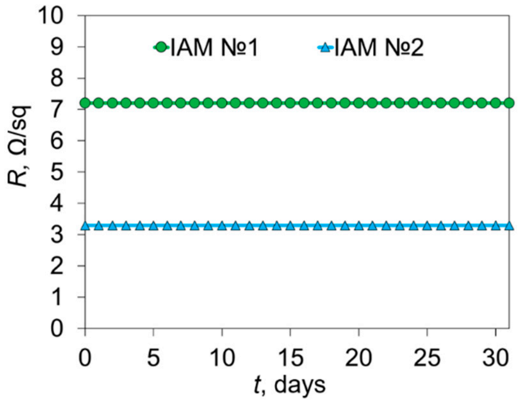

Figure S4 IAM №1 and IAM №2 stability at standard condition

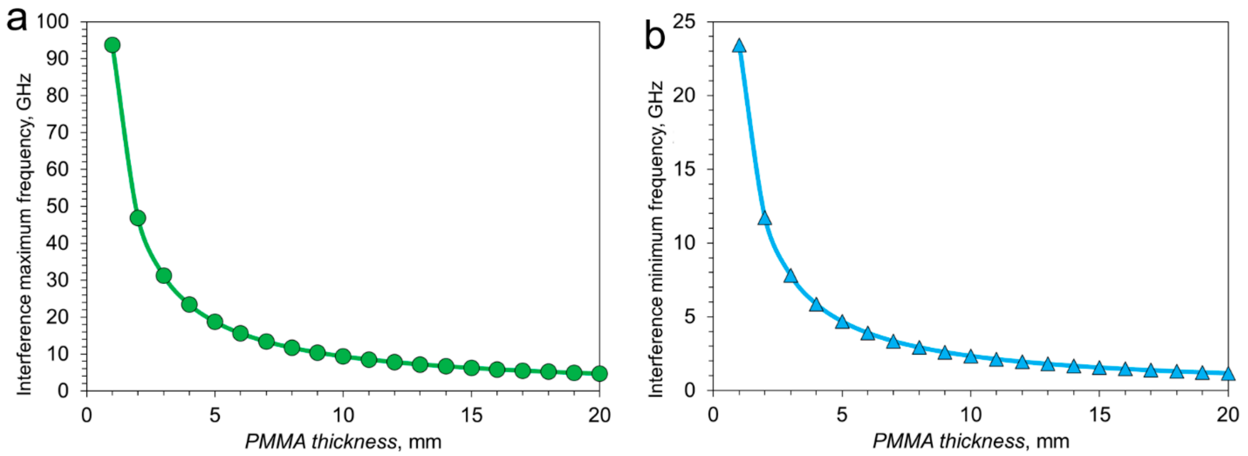

Figure S5 Dependence of the interference maximum (a) and minimum (b) frequency of the studied sandwich structures on the thickness of the PMMA spacer
